# Conservation genetics in Chinese sheep: diversity of fourteen indigenous sheep (*Ovis aries*) using microsatellite markers

**DOI:** 10.1002/ece3.1891

**Published:** 2016-01-18

**Authors:** Guang‐Xin E, Tao Zhong, Yue‐Hui Ma, Hui‐Jiang Gao, Jian‐Ning He, Nan Liu, Yong‐Ju Zhao, Jia‐Hua Zhang, Yong‐Fu Huang

**Affiliations:** ^1^ College of Animal Science and TechnologyChongqing Key Laboratory of Forage & HerbivoreChongqing Engineering Research Centre for Herbivores Resource Protection and UtilizationSouthwest UniversityChongqing400716China; ^2^ Farm Animal Genetic Resources Exploration and Innovation Key Laboratory of Sichuan provinceSichuan Agricultural UniversityChengduSichuan625014China; ^3^ Institute of Animal ScienceChinese Academy of Agricultural Sciences (CAAS)Beijing100193China; ^4^ College of Animal Science and TechnologyQingdao Agricultural UniversityQingdao266109China

**Keywords:** China, Diversity, indigenous sheep, microsatellite

## Abstract

The domestic sheep (*Ovis aries*) has been an economically and culturally important farm animal species since its domestication around the world. A wide array of sheep breeds with abundant phenotypic diversity exists including domestication and selection as well as the indigenous breeds may harbor specific features as a result of adaptation to their environment. The objective of this study was to investigate the population structure of indigenous sheep in a large geographic location of the Chinese mainland. Six microsatellites were genotyped for 611 individuals from 14 populations. The mean number of alleles (±SD) ranged from 7.00 ± 3.69 in Gangba sheep to 10.50 ± 4.23 in Tibetan sheep. The observed heterozygote frequency (±SD) within a population ranged from 0.58 ± 0.03 in Gangba sheep to 0.71 ± 0.03 in Zazakh sheep and Minxian black fur sheep. In addition, there was a low pairwise difference among the Minxian black fur sheep, Mongolian sheep, Gansu alpine merino, and Lanzhou fat‐tailed sheep. Bayesian analysis with the program STRUCTURE showed support for 3 clusters, revealing a vague genetic clustering pattern with geographic location. The results of the current study inferred high genetic diversity within these native sheep in the Chinese mainland.

## Introduction

The domestic sheep (*Ovis aries*) has been an economically and culturally important farm animal species worldwide, since domestication. However, commercial lines and industrialized livestock production systems have spread over all continents resulting in decreasing of large indigenous sheep breeds in comparison with some commercial breeds. Many studies have assessed the diversity of native local sheep in India (Dorji et al. 2010; Pandey et al. 2010; Arora et al. 2011), the Middle East and Europe (Alvarez et al. 2005; Lawson Handley et al. 2007; Peter et al. 2007; Zahedi‐Zahra et al. 2007; Dalvit et al. 2009; Glowatzki‐Mullis et al. 2009; Bowles et al. 2014; Yilmaz et al. 2014; Pons et al. 2015), Eurasia (Blackburn et al. 2011a; Paiva et al. 2011a,b; Salamon et al. 2014), America (Blackburn et al. 2011b; Paiva et al. 2011a,b; Souza et al. 2012; Crispim et al. 2013; Ferreira et al. 2014), and Africa (Gizaw et al. 2007; Agaviezor et al. 2012; Qwabe et al. 2013; Gaouar et al. 2015).

In recent years, several microsatellite studies on diversity in Chinese sheep have been published (Jia et al. 2003; Gao and Wu 2005; Yuan et al. 2006; Sun et al. 2007; Zhong et al. 2011). However, these studies primarily considered a relatively small group of breeds. The Chinese mainland is a rich source of diverse ovine germplasm and contains 67 million sheep that belong to 42 described indigenous breeds (China National Commission of Animal Genetic Resources, 2011). This represents selection by man as well as the adaptation of sheep to different nutrient supplies and climates in China, which is a geographically complex continent and includes areas such as the Tibetan plateau regions. Currently, the number of breeds is rapidly decreasing because of increases in agriculture, industrialization, the no availability of proven rams, shifts in profession and the absence of any planned strategies for their conservation.

The objective of this study was to assess the genetic diversity and breed structure of fourteen Chinese local breeds, with the ultimate aim of maintaining and conserving those breeds. The results of this study allow us to have an idea about the genetic diversity and phylogenetic relationships between the studied breeds.

## Material and Methods

### Animals and experimental methods

We genotyped 611 individuals from 14 breeds from different geographic locations in the Chinese mainland (Table 1). Individuals were genotyped at the six microsatellite loci (Kappes et al. 1997; Maddox et al. 2001 and FAO 2011) that were suggested for biodiversity studies in sheep (Table 2). The methods of DNA extracted and the PCR protocols reference as Zhong et al. (2011). Approximately, 1–2 μL of PCR product was diluted with 10 μL of autoclaved distilled water for use in DNA genotyping. Two microliters of diluted products were added to 7.75 μL Hi Di^™^ formamide and 0.25 Gene Scan‐500 LIZ^™^ (Applied Bio systems, USA). The mixtures were heated at 94°C for 5 min and then immediately chilled on ice for 2 min. Genotyping was performed on a Genetic Analyzer 3130 xl (Applied Bio systems, USA).

**Table 1:** Sampling information of 14 native sheep in China

Name	Code	SZ	N	E	Location
Tibetan sheep	TS	32	29°46′48.56″	94°22′21.49″	Ling Zhi, Tibetan
ZhaoTong sheep	ZT	48	27°20′17.65″	103°42′59.00″	Zhao Tong, YunNan
Anduo sheep	AD	47	33°19′4.83″	90°33′41.33″	AnDuo, Tibetan
Zazakh	HZK	42	42°20′13.92″	93°31′16.51″	Hami, XinJiang
Hu sheep	HU	48	31°18′50.01″	120°36′33.48″	SuZhou, ZheJiang
hulunber	HBR	48	49°11′36.00″	119°44′49.59″	Hulunber, Inner Mongolian
Small‐tailed Han	STH	48	35°15′23.44″	115°27′3.60″	HeZe, ShanDong
Tan sheep	TAN	48	37°37′6.05″	107°02′18.24″	YanChi, NingXia
Gangba sheep	GB	44	28°18′51.22″	88°33′48.37″	GangBa, Tibetan
Ujumqin	UQ	48	44°04′14.47″	116°07′24.96″	Xilihaote, Inner Mongolian
Minxian black sheep fur	MXB	40	34°25′30.71″	104°14′15.50″	Minxian, Gansu
Mongolian sheep	MGH	40	49°16′16.81″	120°01′44.86″	Hailaer, Inner Mongolian
Gansu alpine merino	GSH	40	38°55′56.72″	100°27′6.38″	Zhangye, GanSu
Lanzhou fat‐tailed sheep	LZD	38	36°03′29.71″	103°48′51.92″	LanZhou, Gansu

SZ is Sample size, N is North latitude, E is East longitude, Code is short name of breed.

**Table 2:** Primer information of six microsatellites in current study

Locus	Chro.	Reference	TM(°C)	Sequences
MCM527	OAR 5	Maddox et al. (2001)	56	F:5′‐ GTCCATTGCCTCAAATCAAATTC‐3′
				R:5′‐AAACCACTTGACTACTCCCCAA‐3′
ILSTS005	BTA 10	Kappes et al. (1997)	55	F:5′‐ GGAAGCAATGAAATCTATAGCC‐3′
		Maddox et al. (2001)		R:5′‐TGTTCTGTGAGTTTGTAAGC‐3′
MAF209	OAR 17	Maddox et al. (2001)	65	F:5′‐GATCACAAAAAGTTGGATACAACCGTGG‐3′
				R:5′‐TCATGCACTTAAGTATGTAGGATGCTG‐3′
OarJMP29	OAR 24	Maddox et al. (2001)	65	F: 5′‐GTATACACGTGGACACCGCTTTGTAC‐3′
				R:5′‐GAAGTGGCAAGATTCAGAGGGGAAG‐3′
OarAE129	OAR 5	Kappes et al. (1997)	60	F:5′‐AATCCAGTGTGTGAAAGACTAATCCAG‐3′
		Maddox et al. (2001)		R:5′‐GTAGATCAAGATATAGAATATTTTTCAACACC‐3′
OarFCB304	OAR 19	Kappes et al. (1997)	60	F:5′‐CCCTAGGAGCTTTCAATAAAGAATCGG‐3′
		Maddox et al. (2001)		R:5′‐CGCTGCTGTCAACTGGGTCAGGG‐3′

Chro is the Chromosomal location of microsatellite.

### Data analysis

Genetic diversity expected (*H*_E_), observed (*H*_O_) heterozygosity, mean number of alleles (*N*_A_), and polymorphism information content (PIC) were estimated from the allele frequencies using FSTAT 2.9.3.2 (Goudet 1995). For each locus‐breeds combination of the global data set and breeds groupings, we used Fisher's exact test with Bonferroni correction to test possible deviations from Hardy–Weinberg equilibrium (HWE) using GENEPOP 3.4 (Raymond and Rousset 1995). Pairwise differences in the populations (*F*_ST_, Slatkin 1995) were displayed using the Arlequin software 3.5.1.3 (Excoffier and Lischer 2010). The Bayesian clustering algorithm was implemented in STRUCTURE 2.3.3 (Pritchard et al. 2000; Falush et al. 2003) to determine the population structure and to explore the assignment of individuals and populations to specific gene clusters using a burn‐in of 50,000 followed by 100,000 Markov Chain Monte Carlo (MCMC) iterations from *K*2 to *K*14, in 50 iterations. STRUCTURE_Harvester (Earl and vonHoldt 2012) was used to generate a graphical display of the simulated results and the most optimal *K*. To estimate the most optimal *K*, the number of clusters (*K*) was plotted against Δ*K *= m| *L*′(*K*)|/s|*L*(*K*)|, and the optimal number of clusters was identified by the largest change in the log‐likelihood (*L*(*K*)) values between the estimated number of clusters (Evanno et al. 2005).

## Results

In total, 138 alleles were found in 14 Chinese native sheep breeds across six microsatellite loci. Across breeds, an average of 23 alleles per loci was observed, ranging from 12 in *OarAE129* to 31 in *OarFCB304*. The two extreme loci were *MAF209* with 29 alleles and *OarFCB304* with 31 alleles (Table 3). Across loci, the *N*_A_ ranged from 7.00 ± 3.69 in GB to 10.50 ± 4.23 in TS (Table 4).

**Table 3:** Genetics diversity of all populations by locus

Locus	*H*_O_	*H*_E_	PIC	Na	dHWE
MCM527	0.7647	0.8013	0.7634	22	4
ILSTS005	0.5107	0.5275	0.4824	16	2
MAF209	0.7279	0.7484	0.7134	29	1
OarJMP29	0.7425	0.7484	0.7096	27	1
OarAE129	0.3859	0.5612	0.4897	12	7
OarFCB304	0.6972	0.7287	0.6976	31	2
Mean	0.6382	0.6859	0.6427	23	2.83

dHWE is number of populations deviated from Hardy–Weinberg equilibrium.

**Table 4:** Polymorphism measures for 14 sheep populations

Pop	*H*_O_ (±SD)	*H*_E_ (±SD)	*N*_A_ (±SD)	*F*_IS_	*P*‐Value	dHWE	Pa
TS	0.62 ± 0.04	0.73 ± 0.07	10.50 ± 4.23	0.16	0.0006^a^	3	9
ZT	0.60 ± 0.03	0.72 ± 0.02	8.83 ± 2.79	0.17	0.0006^a^	1	2
AD	0.60 ± 0.03	0.67 ± 0.07	8.00 ± 2.97	0.11	0.0012	1	–
HZK	0.71 ± 0.03	0.73 ± 0.05	9.33 ± 3.56	0.02	0.2667	1	–
HU	0.66 ± 0.03	0.68 ± 0.04	7.17 ± 2.23	0.03	0.1827	1	–
HBR	0.68 ± 0.03	0.71 ± 0.05	9.67 ± 3.08	0.05	0.0720	0	1
STH	0.67 ± 0.03	0.70 ± 0.05	8.33 ± 3.27	0.04	0.0964	1	1
TAN	0.61 ± 0.03	0.66 ± 0.07	8.67 ± 3.39	0.08	0.0119	1	–
GB	0.58 ± 0.03	0.65 ± 0.05	7.00 ± 3.69	0.12	0.0185	2	–
UQ	0.60 ± 0.03	0.65 ± 0.06	9.00 ± 3.22	0.08	0.0143	0	–
MXB	0.71 ± 0.03	0.71 ± 0.06	7.83 ± 2.93	0.00	0.5298	1	3
MGH	0.60 ± 0.03	0.64 ± 0.05	7.50 ± 1.87	0.08	0.0286	1	1
GSH	0.69 ± 0.03	0.72 ± 0.03	7.67 ± 1.75	0.05	0.1042	2	1
LZD	0.60 ± 0.03	0.61 ± 0.06	7.17 ± 1.17	0.01	0.3542	2	–

Pa is number of private allele, dHWE is number of populations deviated from Hardy–Weinberg equilibrium.

a Indicative adjusted nominal level (5%) for one table is 0.0006 based on 1680 randomisations of *P*‐value for *F*_IS_.

The mean observed and expected heterozygote frequencies within loci across the breed was 0.6382 (0.3859 to 0.7647) and 0.6859 (0.5275 to 0.8013), respectively (Table 3). The average polymorphism information content across loci was 0.6427 and ranged from 0.4824 (*ILSTS005*) to 0.7634 (*MCM527*) among breeds (Table 3). Across loci, the *H*_E_ within a breed ranged from 0.61 ± 0.06 in LZD to 0.73 ± 0.07 in TS. The *H*_O_ ranged from 0.58 ± 0.03 in GB to 0.71 ± 0.03 in HZK and MXB (Table 4).

For the Hardy–Weinberg equilibrium, on average, each locus deviated from HWE in 2.83 breeds. The most extreme locus, *MCM527*, deviated from HWE in four breeds (Table 3) and OarAE129 with 7. The UQ and HBR were at HWE for all loci, and at the other extreme, the TS deviated from HWE at 3 loci (Table 4).

The range of the inbreeding coefficient (*F*_IS_) within a breed range from 0.00 was MXB to 0.17 was ZT. It was below 0.1 in ten breeds and above this value in 4 breeds (ZT, TS, AD, and GB). There were two breeds (ZT and TS) carried the *P*‐value of inbreeding coefficients are significantly different from zero.

In total, 18 private alleles were distributed across 14 breeds and 6 loci. The frequency of several private alleles within certain breeds was particularly high. For example, the frequency of a private allele (135 bp) at the locus *MAF209* in TS was 20.31% (see Table S1).

**Figure 1:**
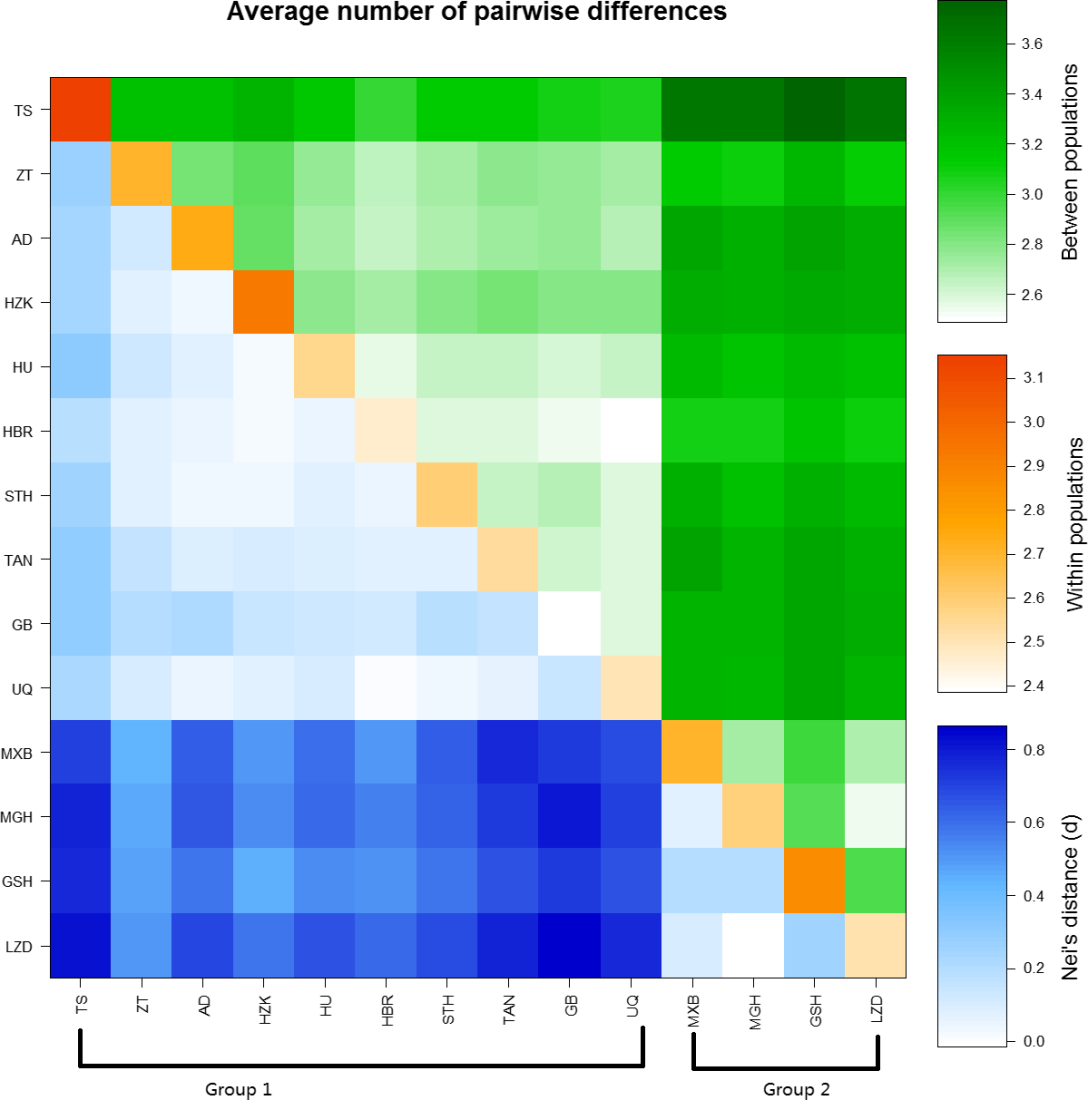
Population average pairwise differences of 14 native Chinese sheep. Above diagonal is average number of pairwise differences between populations, Diagonal elements is average number of pairwise differences within population and below diagonal is corrected average pairwise difference.

In the pairwise difference analysis, the highest diversity within a breed was observed in TS, and the lowest was observed in GB. The group, including GSH, MXB, LED, and MGH, had the lowest difference between breeds compared with the others in the pairwise differences between populations (π*XY*) and consistency to that in corrected average pairwise difference (π*XY*−(π*X* + π*Y*)/2) (Table 5 and Fig. 1).

**Table 5:** Population average pairwise differences of 14 native Chinese sheep

	TS	ZT	AD	HZK	HU	HBR	STH	TAN	GB	UQ	MXB	MGH	GSH	LZD
TS	3.15	3.19*	3.19*	3.29*	3.17*	2.99*	3.13*	3.15*	3.07*	3.05*	3.64*	3.65*	3.77*	3.65*
ZT	0.26*	2.70	2.84*	2.90*	2.76*	2.65*	2.73*	2.78*	2.75*	2.71*	3.14*	3.10*	3.25*	3.11*
AD	0.24*	0.12*	2.74	2.87*	2.72*	2.65*	2.70*	2.73*	2.77*	2.67*	3.36*	3.31*	3.38*	3.32*
HZK	0.24*	0.08*	0.03*	2.94	2.77*	2.72*	2.80*	2.83*	2.81*	2.80*	3.32*	3.29*	3.35*	3.31*
HU	0.31*	0.13*	0.08*	0.03*	2.55	2.56*	2.65*	2.63*	2.60*	2.63*	3.23*	3.17*	3.24*	3.19*
HBR	0.18*	0.07*	0.05*	0.02*	0.05*	2.46	2.58*	2.57*	2.54*	2.49	3.08*	3.08*	3.17*	3.10*
STH	0.26*	0.08*	0.03*	0.03*	0.07*	0.05*	2.59	2.64*	2.68*	2.59*	3.29*	3.21*	3.30*	3.23*
TAN	0.30*	0.16*	0.09*	0.10*	0.08*	0.07*	0.08*	2.54	2.61*	2.59*	3.38*	3.28*	3.37*	3.30*
GB	0.30*	0.20*	0.21*	0.15*	0.13*	0.11*	0.19*	0.15*	2.39	2.58*	3.27*	3.28*	3.35*	3.31*
UQ	0.22*	0.11*	0.05*	0.08*	0.11*	0.01	0.04*	0.07*	0.14*	2.50	3.28*	3.25*	3.35*	3.27*
MXB	0.70*	0.43*	0.64*	0.50*	0.60*	0.50*	0.64*	0.76*	0.72*	0.68*	2.71	2.73*	2.99*	2.71*
MGH	0.78*	0.46*	0.65*	0.53*	0.61*	0.56*	0.62*	0.72*	0.80*	0.71*	0.08*	2.58*	2.92*	2.53
GSH	0.76*	0.47*	0.58*	0.45*	0.53*	0.51*	0.58*	0.67*	0.73*	0.67*	0.20*	0.19*	2.86*	2.93*
LZD	0.82*	0.51*	0.69*	0.59*	0.66*	0.61*	0.68*	0.78*	0.87*	0.77*	0.10*	−0.01	0.25*	2.51

(1) Above diagonal: Average number of pairwise differences between populations (π*XY*); (2) Diagonal elements: Average number of pairwise differences within population (π*X*); (3) Below diagonal: Corrected average pairwise difference (π*XY*−(π*X* + π*Y*); “*” mean the significance *P*‐value (Significance Level = 0.0500) of variance analysis.

**Figure 2:**
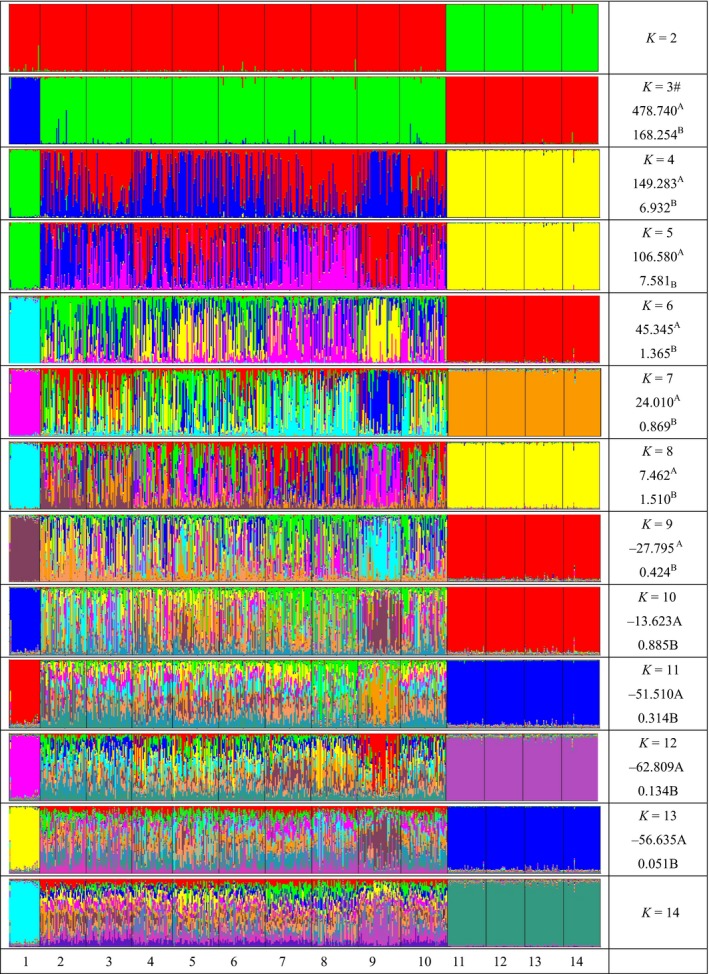
Clustering diagrams of 14 Chinese sheep populations obtained from *K* = 2 to *K* = 14 with best similarities. #label is the most optimal *K*‐value. Note: number of population: TS (1), ZT (2), AD (3), HZK (4), HU (5), HBR (6), STH (7), TAN (8),GB (9), UQ (10), MXB (11), MGH (12), GSH (13), LZD (14). Superscript letter (A) is *L*(*K*), superscript letter (B) is Δ*K* = m| *L*″(*K*)|/s|*L*(*K*)|.

The STRUCTURE software was used for clustering individuals into 2 ≤ *K *≤* *14. At the lowest *K*‐value (*K *=* *2), the MXB, MGH, GSH, and LZD breeds split from the others to form their own cluster. At *K *=* *3 to *K *=* *14, the TS separated and formed an independent cluster base on the clustering diagrams of *K*2, the optimal *K*‐value was thus 3 (Fig. 2).

## Discussion

The results obtained in a previous study for *H*_E_ (ranging from 0.62 to 0.71), *H*_O_ (ranging from 0.65 to 0.69), and *N*_A_ (ranging from 5.22 ± 1.67 to 8.92 ± 3.20) in Mongolian sheep (Zhong et al. 2011) are consistent with those obtained in the current study. These six highly polymorphic microsatellite loci selected in this study allow us to present a general genetic pattern and the phlylogenetic relationship of these breeds.

Deviations from HWE are expected if individual populations are substructured into flocks within populations that are isolated from each other or if inbreeding has occurred in the population (Granevitze et al. 2007). In this study, TS has the largest number (3) of loci that deviated from HWD, and the high *N*_A_ and relatively low *H*_O_ are due to the high diversity within this population. But this is excepted if individual populations are substructured into flocks within populations that are isolated from each other, or if inbreeding has occurred in the populations as while. In addition, higher *F*_IS_ value (0.16) in TS also explains the deficiency of heterozygotes in this population that deviate from HWD.

However, for most populations, the *H*_E_ and *H*_O_ were consistent, and the *F*_IS_ of 12 of 14 breeds was not significantly different from zero in this study, which suggests that most of these indigenous breeds are close to the Hardy–Weinberg equilibrium state.

The pairwise difference, *F*_ST_ value that was observed between some populations (LZD, MGH, GSH, and MXB), was generally lower than that observed between other breeds, thus indicating moderate‐to‐high genetic similarity in this subpopulation (Group 2). For the other subpopulation (Group 1), the high genetic differences indicated a more complex genetic background and different artificial selection direction during their domestication.

The STRUCTURE analysis (Fig. 1) showed a clear clustering of these indigenous sheep and was consistent with the pairwise *F*_ST_ value analysis described above (Fig. 1). For *K *=* *3 to *K *=* *14, the TS was independently clustered, and the Group 1 breeds (excluding TS) and Group 2 breeds were separated into their own clusters. In addition, the background of Group 1 was increasingly complex with increasing *K*‐value, similar to the result of the pairwise *F*_ST_ value, which indicates that gene flow exists in exchange or during multi‐complex ancient domestication. Gene flow between breeds can also be assessed by the abundance of a private allele (Slatkin and Barton 1989; and Granevitze et al. 2007). Therefore, the breed TS, which had the largest number of private alleles, with nine, was likely the first to split from the other breeds. Chinese indigenous sheep including three main pedigrees, such as Tibetan group, Mongolian group, and Kazak group. Their relative species are Urial (*Ovis vignei*) and Agarl (*Ovis ammon*). In addition, the ancestor of Tibetan sheep was demisted from *Ovis vignei* which living in Qinghai–Tibetan Plateau. However, Mongolian group sheep were derived from argali in central Asian mountains region (China National Commission of Animal Genetic Resources. 2011). Therefore, the different ancestor would create their different population structure and diversity level, too.

The optimal *K*‐value was found to be 3 in STRUCTURE clustering. For *K *=* *3, three of the Group 2 breed (MXB, GSH, and LZD) were bred in Gansu Province, and one (MGH) was from Mongolian. This result suggests that the Gansu breeds and Mongolian sheep are indistinguishable, though they were separate for many hundreds of years at domestication sites and have different phenotypes. There may have been some gene flow between them in the past or shared ancestors. For a similar case, the Group 1 breeds, which represents an independent cluster, had a breed that was sampled over a large geographic region in the Chinese mainland and were not only separated into independent clusters but also carried a common large‐complex genetic background, which indicated the general exchange of genetic material. The strong gene flow among regions induced by human migration, commercial trade, and the extensive transport of sheep was identified by the variability of mtDNA (Zhao et al. 2013) in China. Therefore, we could not conclude that there were two domestication sites or shared common ancestors in the China mainland according to the clustering diagrams. Thus, obtaining additional direct evidence from different regions is necessary and should include disciplines such as archeology. However, from the clustering analysis and genetic diversity state, particularly the private alleles in the TS breed and other Tibetan breeds, it possible that there were more than two domestication sites of Tibetan region sheep in this study. However, this study only presents a general idea or retrieves a rough idea of genetic pattern and diversity status in those Chinese indigenous sheep. Therefore, in further study a more subtle population structure might be revealed using more genetic markers.

In short, six microsatellites were genotyped for 611 individuals from 14 breeds to investigate the breed structure of indigenous sheep in China. The results of the current study infer affluent genetic diversity within breeds and strong gene flow exchange between native sheep in the Chinese mainland.

## Conflicts of Interest

None declared.

## Supporting information


**Table S1.** Allelic frequency of six microsatellite in each population.
