# Supplementary material for: Conservation genetics in Chinese sheep: diversity of fourteen indigenous sheep (Ovis aries) using microsatellite markers
Source: Ecol Evol. 2016 Jan 18;6(3):810–7. doi: 10.1002/ece3.1891 (PMC4739567; doi:10.1002/ece3.1891)
Supplement: Supplementary file 1 — Table S1. Allelic frequency of six microsatellite in each population. [file ECE3-6-810-s001.doc]

Supplemental material Table 1.Allelic frequency of six microsatellite in each population

a.Allelic frequency of MCM527

|  | TS | ZT | AD | HZK | HU | HBR | STH | TAN | GB | UQ | MXB | MGH | GSH | LZD |
| --- | --- | --- | --- | --- | --- | --- | --- | --- | --- | --- | --- | --- | --- | --- |
| 161 |  |  |  | 3.75 |  | 1.16 |  |  |  |  |  |  |  |  |
| 163 |  |  |  | 3.75 |  | 1.16 |  |  |  | 1.09 |  |  |  |  |
| 164 | 1.56 |  |  |  |  |  |  |  |  |  | 6.33 |  |  |  |
| 165 | 1.56 | 3.41 |  |  |  | 3.49 |  | 1.04 |  |  |  |  |  |  |
| 166 | 10.94 |  |  |  |  |  |  |  |  |  | 24.05 | 30.00 | 11.25 | 27.63 |
| 167 | 12.50 | 5.68 | 29.55 | 18.75 | 22.83 | 32.56 | 25.00 | 21.88 | 16.22 | 38.04 |  |  |  |  |
| 168 |  |  |  |  |  |  |  | 1.04 |  |  | 21.52 | 26.25 | 32.50 | 32.89 |
| 169 | 31.25 | 31.82 | 22.73 | 28.75 | 15.22 | 15.12 | 36.46 | 17.71 | 16.22 | 22.83 |  |  |  |  |
| 170 |  |  |  |  |  |  |  |  |  |  | 7.59 | 6.25 | 21.25 | 2.63 |
| 171 | 6.25 | 19.32 | 14.77 | 12.50 | 2.17 | 11.63 | 5.21 | 6.25 | 6.76 | 7.61 |  |  |  |  |
| 172 |  |  |  |  |  |  |  |  |  |  | 3.80 | 15.00 | 3.75 | 22.37 |
| 173 | 9.38 | 15.91 | 12.50 | 5.00 | 10.87 | 11.63 | 17.71 | 4.17 | 6.76 | 13.04 |  |  |  |  |
| 174 |  |  |  |  |  |  |  |  |  |  | 26.58 | 11.25 | 28.75 | 7.89 |
| 175 | 10.94 | 11.36 | 9.09 | 17.50 | 35.87 | 19.77 | 12.50 | 22.92 | 41.89 | 11.96 |  |  |  |  |
| 176 |  |  |  |  |  | 1.16 |  |  |  |  | 1.27 | 5.00 | 1.25 | 2.63 |
| 177 | 6.25 | 11.36 | 5.68 |  | 4.35 |  |  | 8.33 | 1.35 |  |  |  |  |  |
| 178 |  |  |  |  |  |  |  |  |  |  |  | 2.50 | 1.25 |  |
| 179 | 7.81 | 1.14 | 4.55 | 1.25 | 1.09 | 1.16 |  | 2.08 |  | 2.17 |  |  |  |  |
| 180 |  |  |  |  |  |  |  |  |  |  | 5.06 | 1.25 |  | 1.32 |
| 181 | 1.56 |  | 1.14 | 6.25 | 7.61 |  | 3.13 | 13.54 | 9.46 | 1.09 |  |  |  |  |
| 182 |  |  |  |  |  |  |  |  |  |  | 3.80 | 2.50 |  | 2.63 |
| 183 |  |  |  | 2.50 |  | 1.16 |  | 1.04 | 1.35 | 2.17 |  |  |  |  |

Note: first column is the sequence size of allele (bp)

b.Allelic frequency of ILSTS005

|  | TS | ZT | AD | HZK | HU | HBR | STH | TAN | GB | UQ | MXB | MGH | GSH | LZD |
| --- | --- | --- | --- | --- | --- | --- | --- | --- | --- | --- | --- | --- | --- | --- |
| 186 |  | 1.09 |  | 1.25 |  | 1.28 |  |  |  |  |  |  |  |  |
| 188 |  | 4.35 |  |  |  |  | 2.08 |  |  |  |  |  | 5.00 |  |
| 190 | 9.38 | 2.17 | 1.14 | 3.75 |  | 5.13 |  | 1.04 | 1.19 | 2.17 |  |  | 1.25 |  |
| 192 |  |  |  |  |  |  |  |  |  |  |  | 5.00 | 2.50 | 1.32 |
| 194 | 57.81 | 51.09 | 71.59 | 55.00 | 58.70 | 65.38 | 65.63 | 78.13 | 69.05 | 75.00 |  |  | 1.25 |  |
| 195 | 3.13 |  |  |  |  |  |  |  |  |  |  |  |  |  |
| 196 | 10.94 | 25.00 | 10.23 | 11.25 | 8.70 | 7.69 | 13.54 | 5.21 | 2.38 | 7.61 | 68.75 | 71.25 | 36.25 | 77.63 |
| 198 |  | 1.09 |  |  |  |  | 1.04 |  |  | 3.26 | 7.50 | 10.00 | 28.75 | 3.95 |
| 200 |  |  |  | 1.25 | 4.35 | 1.28 | 3.13 |  | 2.38 | 1.09 |  |  | 3.75 |  |
| 201 | 15.63 |  |  |  |  |  |  |  |  |  |  |  |  |  |
| 202 |  | 15.22 | 15.91 | 22.50 | 25.00 | 15.38 | 13.54 | 14.58 | 25.00 | 6.52 | 23.75 | 13.75 | 20.00 | 14.47 |
| 203 | 1.56 |  |  |  |  |  |  |  |  |  |  |  |  |  |
| 204 |  |  | 1.14 | 1.25 | 3.26 | 1.28 |  | 1.04 |  | 1.09 |  |  |  |  |
| 210 |  |  |  |  |  |  | 1.04 |  |  |  |  |  |  | 2.63 |
| 214 | 1.56 |  |  |  |  |  |  |  |  |  |  |  |  |  |
| 216 |  |  |  | 3.75 |  | 2.56 |  |  |  | 3.26 |  |  | 1.25 |  |

Note: first column is the sequence size of allele (bp)

c.Allelic frequency of MAF209

|  | TS | ZT | AD | HZK | HU | HBR | STH | TAN | GB | UQ | MXB | MGH | GSH | LZD |
| --- | --- | --- | --- | --- | --- | --- | --- | --- | --- | --- | --- | --- | --- | --- |
| 104 |  |  |  |  |  |  |  |  |  |  | 1.25 |  |  |  |
| 105 |  | 5.95 | 2.44 |  |  | 1.39 |  |  | 3.41 |  |  |  |  |  |
| 106 |  |  |  |  |  |  |  |  |  |  | 18.75 | 8.75 | 7.50 | 7.89 |
| 107 |  |  | 1.22 | 5.95 | 10.00 |  | 9.21 | 9.09 |  | 3.41 |  |  |  |  |
| 111 |  |  |  |  |  |  | 3.95 |  |  | 1.14 |  |  |  |  |
| 112 |  |  |  |  |  |  |  |  |  |  |  | 1.25 |  | 2.63 |
| 114 |  |  |  |  |  |  |  | 1.14 |  |  |  |  |  |  |
| 115 | 1.56 |  | 6.10 | 11.90 | 18.75 | 15.28 | 9.21 | 19.32 | 11.36 | 13.64 |  |  |  |  |
| 116 | 1.56 |  |  |  |  |  |  | 1.14 |  |  | 5.00 | 3.75 | 5.00 | 1.32 |
| 117 |  | 45.24 | 58.54 | 40.48 | 50.00 | 44.44 | 23.68 | 36.36 | 51.14 | 53.41 |  |  |  |  |
| 118 |  |  |  |  |  |  |  |  |  |  | 30.00 | 50.00 | 23.75 | 55.26 |
| 119 |  | 4.76 | 1.22 | 1.19 | 5.00 | 4.17 | 5.26 | 13.64 | 7.95 | 1.14 |  |  |  |  |
| 120 |  |  |  |  |  |  |  |  |  |  | 2.50 | 3.75 |  | 5.26 |
| 121 |  | 1.19 |  | 2.38 |  |  |  |  |  |  |  |  |  |  |
| 122 |  |  |  |  |  |  |  |  |  |  | 3.75 |  |  |  |
| 123 | 3.13 | 9.52 | 15.85 | 2.38 | 3.75 | 2.78 | 1.32 | 4.55 | 9.09 | 1.14 |  |  |  |  |
| 124 |  |  |  |  |  |  |  |  |  |  | 5.00 | 1.25 | 11.25 |  |
| 125 | 21.88 | 14.29 | 3.66 | 5.95 | 6.25 | 4.17 | 10.53 |  | 4.55 | 3.41 |  |  |  |  |
| 126 | 4.69 |  |  |  |  |  |  |  |  |  | 8.75 | 6.25 | 13.75 | 7.89 |
| 127 | 3.13 | 4.76 | 8.54 | 17.86 | 3.75 | 15.28 | 23.68 | 10.23 | 10.23 | 19.32 |  |  |  |  |
| 128 |  |  |  |  |  |  |  |  |  |  | 20.00 | 21.25 | 30.00 | 14.47 |
| 129 | 3.13 | 9.52 | 2.44 | 11.90 | 2.50 | 8.33 | 10.53 | 1.14 | 2.27 | 3.41 |  |  |  |  |
| 130 |  |  |  |  |  |  |  |  |  |  | 5.00 | 3.75 | 6.25 | 5.26 |
| 131 | 6.25 | 4.76 |  |  |  | 4.17 | 2.63 | 2.27 |  |  |  |  |  |  |
| 132 |  |  |  |  |  |  |  |  |  |  |  |  | 2.50 |  |
| 133 | 28.13 |  |  |  |  |  |  | 1.14 |  |  |  |  |  |  |
| 135 | 20.31 |  |  |  |  |  |  |  |  |  |  |  |  |  |
| 137 | 3.13 |  |  |  |  |  |  |  |  |  |  |  |  |  |
| 144 | 3.13 |  |  |  |  |  |  |  |  |  |  |  |  |  |

Note: first column is the sequence size of allele (bp)

d.Allelic frequency of OarJMP29

|  | TS | ZT | AD | HZK | HU | HBR | STH | TAN | GB | UQ | MXB | MGH | GSH | LZD |
| --- | --- | --- | --- | --- | --- | --- | --- | --- | --- | --- | --- | --- | --- | --- |
| 116 |  | 3.13 |  |  |  |  |  |  |  |  |  |  |  |  |
| 118 |  | 2.08 | 7.45 |  |  | 2.17 | 1.14 |  |  |  |  |  | 1.25 |  |
| 120 | 3.13 |  |  |  |  |  |  |  |  |  |  |  |  |  |
| 123 | 3.13 |  |  |  |  |  |  |  |  |  |  |  |  |  |
| 124 |  |  |  |  |  |  |  | 1.04 |  |  |  |  |  |  |
| 125 | 23.44 |  |  |  |  |  |  |  |  |  |  |  |  |  |
| 126 | 1.56 |  |  |  |  |  |  |  |  |  |  |  |  |  |
| 127 | 1.56 |  |  |  |  |  |  |  |  |  |  |  |  |  |
| 128 |  | 3.13 | 3.19 | 5.95 | 1.16 | 3.26 | 6.82 |  |  | 1.09 |  | 3.75 | 2.50 |  |
| 129 | 3.13 |  |  |  |  |  |  |  |  |  |  |  |  |  |
| 130 | 1.56 |  | 15.96 | 13.10 | 12.79 | 9.78 | 11.36 | 13.54 | 13.64 | 13.04 | 6.41 | 16.25 | 11.25 | 15.79 |
| 131 | 4.69 |  |  |  |  |  |  |  |  |  |  |  |  |  |
| 132 |  | 3.13 | 10.64 | 2.38 | 3.49 | 3.26 | 4.55 |  |  | 5.43 | 3.85 | 1.25 |  | 3.95 |
| 133 | 25.00 |  |  |  |  |  |  |  |  |  |  |  |  |  |
| 134 |  |  |  | 1.19 |  |  |  |  | 1.14 |  |  |  |  |  |
| 135 | 17.19 |  |  |  |  |  |  |  |  |  |  |  |  |  |
| 136 | 3.13 | 11.46 |  | 2.38 | 2.33 | 7.61 |  | 2.08 |  | 9.78 | 2.56 | 1.25 | 6.25 | 5.26 |
| 137 | 6.25 |  |  |  |  |  |  |  |  |  |  |  |  |  |
| 138 |  | 34.38 | 41.49 | 44.05 | 51.16 | 27.17 | 48.86 | 42.71 | 11.36 | 27.17 | 30.77 | 50.00 | 57.50 | 48.68 |
| 140 |  | 35.42 | 11.70 | 16.67 | 15.12 | 27.17 | 19.32 | 12.50 | 29.55 | 28.26 | 34.62 | 21.25 | 12.50 | 18.42 |
| 142 |  | 5.21 | 5.32 | 10.71 | 6.98 | 6.52 | 6.82 | 4.17 | 37.50 | 8.70 | 3.85 | 2.50 | 8.75 |  |
| 143 | 1.56 |  |  |  |  |  |  |  |  |  |  |  |  |  |
| 144 | 4.69 |  |  |  |  |  |  | 3.13 |  |  |  |  |  |  |
| 146 |  | 2.08 | 4.26 | 1.19 | 6.98 | 10.87 | 1.14 | 13.54 | 6.82 | 4.35 |  |  |  | 2.63 |
| 148 |  |  |  |  |  | 1.09 |  |  |  | 1.09 | 8.97 | 3.75 |  | 5.26 |
| 150 |  |  |  | 2.38 |  |  |  | 7.29 |  | 1.09 | 8.97 |  |  |  |
| 152 |  |  |  |  |  | 1.09 |  |  |  |  |  |  |  |  |

Note: first column is the sequence size of allele (bp)

e.Allelic frequency of OarJMP29

|  | TS | ZT | AD | HZK | HU | HBR | STH | TAN | GB | UQ | MXB | MGH | GSH | LZD |
| --- | --- | --- | --- | --- | --- | --- | --- | --- | --- | --- | --- | --- | --- | --- |
| 135 | 10.94 |  |  |  |  |  |  |  |  |  |  |  |  |  |
| 137 |  | 10.26 | 11.11 |  | 1.22 | 5.81 | 15.22 | 23.40 |  | 7.81 | 6.58 | 2.50 | 2.56 | 1.35 |
| 139 |  | 1.28 |  |  |  |  |  |  |  |  | 2.63 |  |  |  |
| 141 |  |  |  |  |  |  |  |  |  |  |  |  | 2.56 | 1.35 |
| 145 |  | 6.41 | 4.17 | 1.39 |  | 1.16 |  |  |  |  |  | 1.25 |  | 1.35 |
| 146 | 12.50 |  |  |  |  |  |  |  |  |  |  |  |  |  |
| 147 |  | 28.21 | 12.50 | 48.61 | 43.90 | 32.56 | 15.22 | 7.45 | 61.11 | 23.44 | 32.89 | 26.25 | 47.44 | 20.27 |
| 149 | 75.00 | 50.00 | 68.06 | 48.61 | 50.00 | 55.81 | 58.70 | 53.19 | 38.89 | 65.63 | 52.63 | 58.75 | 33.33 | 70.27 |
| 151 | 1.56 | 3.85 | 4.17 | 1.39 | 4.88 | 4.65 | 9.78 | 15.96 |  | 3.13 | 3.95 | 5.00 |  | 4.05 |
| 153 |  |  |  |  |  |  |  |  |  |  | 1.32 |  |  |  |
| 163 |  |  |  |  |  |  |  |  |  |  |  | 5.00 | 14.10 |  |
| 169 |  |  |  |  |  |  | 1.09 |  |  |  |  | 1.25 |  | 1.35 |

Note: first column is the sequence size of allele (bp)

f.Allelic frequency of OarFCB304

|  | TS | ZT | AD | HZK | HU | HBR | STH | TAN | GB | UQ | MXB | MGH | GSH | LZD |
| --- | --- | --- | --- | --- | --- | --- | --- | --- | --- | --- | --- | --- | --- | --- |
| 142 | 1.56 |  |  |  |  |  |  |  |  |  |  |  |  |  |
| 144 | 4.69 |  |  |  |  |  |  |  |  |  |  |  |  |  |
| 147 |  |  |  |  |  |  | 12.77 |  |  | 2.17 |  |  |  |  |
| 149 | 1.56 | 3.19 | 13.83 | 3.57 |  | 3.26 | 1.06 | 4.17 | 3.49 | 1.09 |  | 2.50 |  | 5.26 |
| 151 |  | 1.06 |  |  | 1.06 | 2.17 |  |  |  |  |  |  | 2.50 |  |
| 153 | 6.25 |  | 6.38 | 5.95 |  | 1.09 | 2.13 | 1.04 | 4.65 | 1.09 |  |  |  |  |
| 155 |  |  |  |  |  | 1.09 |  | 2.08 |  | 3.26 |  |  |  |  |
| 157 |  |  | 3.19 |  |  |  |  |  |  | 3.26 |  |  |  |  |
| 159 | 7.81 |  | 4.26 |  |  |  |  |  |  | 1.09 |  |  |  |  |
| 161 | 1.56 | 2.13 | 2.13 | 1.19 |  |  |  |  | 2.33 |  |  |  |  |  |
| 163 | 12.50 | 3.19 | 4.26 | 11.90 | 6.38 | 17.39 | 1.06 | 6.25 | 1.16 | 8.70 | 21.79 | 6.25 | 5.00 | 7.89 |
| 164 | 6.25 | 1.06 |  |  |  |  |  |  | 17.44 |  |  |  |  |  |
| 165 | 39.06 | 52.13 | 32.98 | 34.52 | 40.43 | 45.65 | 48.94 | 65.63 | 50.00 | 52.17 | 41.03 | 58.75 | 41.25 | 55.26 |
| 167 | 1.56 | 3.19 | 8.51 | 8.33 | 4.26 | 4.35 | 3.19 | 1.04 | 1.16 | 3.26 | 12.82 | 8.75 | 1.25 | 10.53 |
| 169 | 1.56 |  | 1.06 | 3.57 |  | 5.43 |  |  | 3.49 |  | 5.13 |  |  |  |
| 171 |  | 14.89 | 10.64 | 2.38 | 5.32 | 5.43 | 7.45 | 1.04 |  | 9.78 |  |  |  |  |
| 172 | 4.69 |  |  |  |  |  |  |  |  |  |  |  |  |  |
| 173 |  | 1.06 |  | 4.76 |  |  | 1.06 | 4.17 | 1.16 |  | 7.69 | 2.50 | 33.75 | 1.32 |
| 174 |  | 1.06 |  |  |  |  |  |  |  |  |  |  |  |  |
| 175 |  | 4.26 |  | 1.19 | 1.06 |  |  |  | 1.16 |  |  |  | 2.50 |  |
| 177 |  | 1.06 | 3.19 |  | 1.06 | 2.17 | 3.19 | 2.08 |  | 1.09 |  |  | 2.50 |  |
| 178 | 7.81 |  |  |  |  |  |  |  |  |  |  |  |  |  |
| 179 |  |  | 6.38 | 5.95 | 13.83 | 3.26 | 11.70 | 2.08 | 1.16 | 6.52 | 1.28 | 3.75 |  |  |
| 180 | 1.56 |  |  |  |  |  |  |  |  |  |  |  |  |  |
| 181 |  | 3.19 | 3.19 | 1.19 |  | 2.17 | 1.06 | 2.08 |  |  | 2.56 | 16.25 | 3.75 | 15.79 |
| 182 | 1.56 |  |  |  |  |  |  |  |  |  |  |  |  |  |
| 183 |  |  |  | 7.14 | 5.32 | 3.26 | 1.06 | 8.33 | 2.33 | 4.35 | 2.56 | 1.25 | 1.25 |  |
| 185 |  |  |  |  |  |  |  |  |  |  | 1.28 |  |  | 2.63 |
| 187 |  | 8.51 |  | 7.14 | 21.28 | 3.26 | 2.13 |  | 10.47 | 2.17 |  |  |  |  |
| 189 |  |  |  | 1.19 |  |  | 3.19 |  |  |  | 2.56 |  |  |  |
| 191 |  |  |  |  |  |  |  |  |  |  | 1.28 |  | 6.25 | 1.32 |
| OarFCB304 | TS | ZT | AD | HZK | HU | HBR | STH | TAN | GB | UQ | MXB | MGH | GSH | LZD |

Note: first column is the sequence size of allele (bp)
